# Recurrent uncomplicated urinary tract infections: definitions and risk factors

**DOI:** 10.3205/id000072

**Published:** 2021-05-27

**Authors:** Tommaso Cai

**Affiliations:** 1 Department of Urology, Santa Chiara Hospital, Trento, Italy

**Keywords:** urinary tract infections, prophylaxis, antibiotics, quality of life, risk factors

## Abstract

**Introduction:** Recurrent uncomplicated urinary tract infections (UTI) have a high impact on patients’ quality of life and high direct and indirect costs for public health. Therefore, optimal management should be of high priority.

**Methods:** Current international guidelines were reviewed, and a systematic literature search was performed in Medline, Cochrane, and Embase.

**Results:** Several risks factors have been identified and used in everyday clinical practice to plan the correct strategy for recurrence prevention. Among all factors, the most important are: sexual intercourse, spermicide use, having a new sex partner, having a mother with a history of UTI, having had UTI during childhood, and asymptomatic bacteriuria treatment. Moreover, other risk factors such as reduced fluid intake, habitual and post-coital delayed urination, wiping from back to front after defecation, douching and wearing occlusive underwear, as well as irregular bowel function should be taken into account.

**Conclusions:** Recurrent UTI show a high impact on clinical practice. Risk factors are generally related to both virulence of pathogens and patient’s behavior or condition. A recently developed nomogram can assist in identifying women at high risk of symptomatic recurrence that can be suitable candidates for a prophylactic strategy.

## Summary of recommendations

1. Recurrent uncomplicated urinary tract infections (rUTI) are defined as at least 3 episodes of a UTI in 12 months, or at least two episodes in 6 months.
2. rUTI have a high impact on public health due to high direct and indirect costs.
3. Sexual intercourse, spermicide use, having a new sex partner, having a mother with a history of UTI, having had UTI during childhood, and asymptomatic bacteriuria treatment are the most important risk factors for rUTI in young women.
4. Atrophic vaginitis due to estrogen deficiency, cystocoele, increased post-void urine volume and functional status deterioration are the most important risk factors in old women.
5. The assessment of all risk factors and the use of a recently validated nomogram could be interesting for identifying women at high risk of symptomatic recurrence that can be suitable candidates for a prophylactic strategy.

## Introduction

Recurrent uncomplicated urinary tract infections (rUTI) may present a big social and economic burden for women of all ages [1]. Therefore, optimal management of rUTI should be of high priority. This review addresses the definitions and the risk factors of recurrent uncomplicated urinary tract infections (rUTI), with particular attention to the following issues: difference between relapse and reinfections, risk factors in premenopausal and postmenopausal women, and the role of a validated nomogram and questionnaires for the calculation of the risk of a new episode of rUTI. The review does not address recurrent UTI in case of significant underlying anatomical or functional abnormalities of the urinary tract, which need a different approach.

## Methods

This review incorporates the 2016 Guidelines on Urological Infections of the European Association of Urology (EAU) and the latest Infectious Diseases Society of America Guideline for the Management of Asymptomatic Bacteriuria [2], [3]. Furthermore, the previous version of the ICUD textbook on Urological infections has been updated and was included [4]. Moreover, a systematic literature search was performed in Medline, Cochrane, and Embase. The systematic literature search covered the period from 1979 to 2019. The following keywords were used: recurrent urinary tract infections, epidemiology, and risk factors. The limitations used included adults aged ≥18 years, clinical studies, review article, English, and peer reviewed. A total of 561 publications were identified and screened by title and abstract. Finally, 7 papers were included in the review (Table 1 (Tab. 1)). The studies were rated according to the level of evidence (LoE) and the grade of recommendation (GoR) using ICUD standards (EAU guidelines 2020 [2]) (Table 1 (Tab. 1)).

## Results

### Definition, context and clinical application

Recurrent urinary tract infections (rUTI) are defined as at least 3 episodes of a UTI in 12 months, or at least 2 episodes in 6 months [1], [2], [3], [5]. Recurrent UTI can be divided into two subgroups:

- relapses
- reinfections.

*Relapse:* is defined as a UTI caused by the same microorganism after adequate treatment [3]. *Reinfection:* is defined as a recurrence of a UTI caused by a different microorganism, or a recurrent UTI caused by a previously isolated microorganism after treatment and a subsequent negative urine culture [3], [5].

This classification shows important clinical issues: relapses signify that the original infection has never been eradicated. The organism cultured is identical to that from the previous episode, and symptoms usually recur within 2 weeks of the end of treatment for the previous episode. If the previous episode was treated with short-course therapy, the first thought should be that there was subclinical pyelonephritis and that a longer course of treatment is needed. If a longer course is followed by another relapse, ‘imaging’ (CT scan or ultrasound) is advisable to look for an anatomic abnormality [6], [7].

### Epidemiology of rUTI

After an initial urinary tract infection, approximately 20–30% of women with a UTI will have a second UTI within 6 months, and 3% will experience a third UTI during that time period [8]. Recurrent UTI are common among young healthy women even though they generally have anatomically and physiologically normal urinary tracts and are associated with considerable morbidity and expense [9], [10]. In detail, it is estimated that in young women overall, there are 0.5 episodes of acute cystitis per person per year, and the incidence decreases with age [11]. In postmenopausal women, it is estimated that there are 0.07 episodes of acute cystitis per person per year [7], [12], [13]. Most recurrences occur within the first 3 months after the primary infection. When the initial infection is caused by *Escherichia coli*, there is a higher risk of reinfection within the first 6 months [13], [14].

### Risk factors

Risk factors are different between young (premenopausal) and old (postmenopausal) women. This difference shows important clinical relevance.

#### Young women

In young healthy women, sexual intercourse is the risk factor most highly associated with recurrent UTI [10]. Other risk factors in young women include spermicide use, having a new sexual partner, having a mother with a history of UTI, and having had a UTI during childhood [15]. There are several behaviors that are thought to increase the risk of recurrent UTI, but their association with UTI has not been clearly demonstrated in trials [10]. These include reduced fluid intake, habitually delaying urination, delaying post-coital urination, wiping from back to front after defecation, douching, and wearing occlusive underwear [10]. Finally, dysfunctional voiding patterns in which there is increased tone of the external sphincter during micturition can also be associated with recurrent UTI in otherwise urologically normal women [15].

##### Sexual intercourse

Any lifetime sexual activity and any sexual activity during the past year were the variables most strongly associated with the risk of recurrence in young women [16]. In detail, sexual intercourse in the past month has been associated with a risk of more than 9 times to develop UTI [OR 10.3 (5.8 to 18.3)] [16]. In particular, young women with recurrent UTI were more likely to report exposure to spermicides and to oral contraceptives [16]. Both intercourse and spermicide exposure increase periurethral *Escherichia coli* colonization, and such colonization occurs more frequently and for prolonged periods in women with rUTI [17], [18].

##### History of UTI in the mother and a history of early UTI onset in the woman herself

The history of UTI in the mother and a history of early UTI onset in the young woman herself were associated with a 2–4-fold increase in the risk of recurrence. These two variables were the most strongly associated with the risk of recurrence, after the strongest variable, recent frequency of sexual intercourse [16]. In particular, Kunin showed that girls who experienced these infections during childhood were more prone to bacteriuria and symptomatic infections as adults [19].

##### Bowel function and water intake

Fecal-perineal-urethral contamination is the most probable explanation for infections caused by enteric bacteria in women, as shown by several authors evaluating the genotype of *Escherichia coli* strains causing UTI in women [20]. Loening-Baucke et al., evaluating a cohort of 234 chronically constipated and encopretic children with a mean follow-up of 15 months, showed that constipation treatment resulted in the disappearance of daytime urinary incontinence in 89%, and night-time urinary incontinence in 63% of patients, as well as disappearance of rUTI in all patients who had no anatomical abnormality of the urinary tract [21]. With regard to water intake, literature data are discordant. Eckford et al. documented a reduction of recurrent UTI in premenopausal women with adequate hydratation with urine osmolality <1105, using an osmolality probe at home, whereas other studies did not find the same correlation [22]. Nygaard et al. surveyed female teachers and found that women who drank less had a 2.21-fold higher risk (95% CI 1.45–3.38) of UTI compared to women who drank the volume they desired at work [23].

##### Asymptomatic bacteriuria treatment

Recently, Cai et al. found that antibiotic treatment of asymptomatic bacteriuria in young women with recurrent UTI is not only unnecessary, but harmful [24], [25]. In fact, they found that in women who had undergone antibiotic treatment, the rate of *E. coli* decreased over time, whereas the prevalence of *E. faecalis* increased gradually, suggesting that *E. faecalis* should be an important defense mechanism that effectively interferes with the establishment of many important enteric pathogens, such as *E. coli* [24].

#### Old women

The incidence of UTI in women increases with advancing age [15]. In a placebo-controlled, double-blind study, Raz et al. showed a correlation between reduced estrogenic hormone levels after menopause and the development of recurrent UTI, highlighting the fact that estrogens stimulate proliferation of *Lactobacillus* in the vaginal epithelium, causing reduction of vaginal pH, thereby preventing vaginal colonization by Enterobacteriaceae [26]. Recently, Lüthje et al. highlighted that estrogen induced the expression of antimicrobial peptides, thereby enhancing the antimicrobial capacity of the urothelium and restricting bacterial multiplication [27]. Furthermore, they suggested the application of estrogen in postmenopausal women suffering from recurrent UTI [27]. Moreover, in older women, risk factors include urinary incontinence, history of UTI before menopause, blood group antigen nonsecretor status, and having a cystocele and an increased post-void residual.

### Nomogram and tools for risk of recurrence calculation

**Figure 1:**
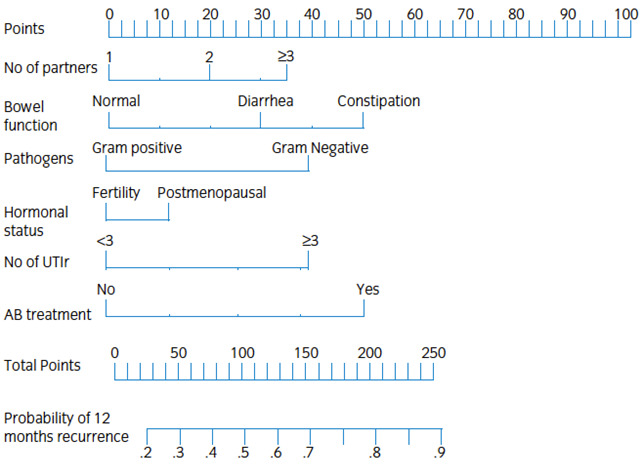
LUTIRE nomogram [27]

Hooton et al. developed a simple risk prediction model by using the information about the number of days with intercourse and contraceptive use (diaphragm and spermicide) for predicting the risk of UTI recurrence [7]. They found that an unmarried, 24-year-old female university student who had sexual intercourse without a diaphragm and spermicide on three of the past seven days had a risk of UTI that was 2.6-fold greater than that of a similar student who had not had intercourse in the previous week [7]. This study highlights the role of recurrence risk prediction tools in the management of women with UTI. In 2014, Cai T et al., for the first time, developed and validated an easy nomogram based on several parameters both from the patients and the bacteria for predicting the recurrence of UTI risk [28]. The nomogram was evaluated by calculating concordance probabilities, as well as testing calibration of predicted urinary tract infection recurrence with observed urinary tract infections. Nomogram variables included: number of partners [29], bowel function, type of pathogens isolated (gram-positive/negative), hormonal status, number of previous urinary tract infection recurrences and previous treatment of asymptomatic bacteriuria. The nomogram accurately predicts the recurrence risk of urinary tract infection at 12 months, and can assist in identifying women at high risk of symptomatic recurrence that can be suitable candidates for a prophylactic strategy (Figure 1 (Fig. 1)) [28]. In order to calculate the recurrence probability, the patient values are identified on each axis, then for each onea vertical line upwards to the “points” axis is drawn. This determines how many points each variable generates. All points for all variables are added, and this sum is placed on the ‘total points’ line. Then a vertical line downwards from this point is drawn, and the recurrence risk probability at 12 months is identified [28].

## Conclusions

Recurrent UTI represent a major social and economic burden for women of all ages. Risk factors for recurrent UTI are generally related to both virulence of pathogens (such as *Escherichia coli* adherent to vaginal and bladder epithelial cells, asymptomatic bacteriuria treatment) and patient’s behavior or condition (such as use of a spermicide or a diaphragm, delayed postcoital micturition, or the ABO-blood-group non-secretor phenotype). A recently developed nomogram can assist in identifying women at high risk of symptomatic recurrence that can be suitable candidates for a prophylactic strategy.

## Note

This article will also be published as a chapter of the Living Handbook “Urogenital Infections and Inflammations” [30].

## Competing interests

The author declares that he has no competing interests.

## Figures and Tables

**Table 1:**
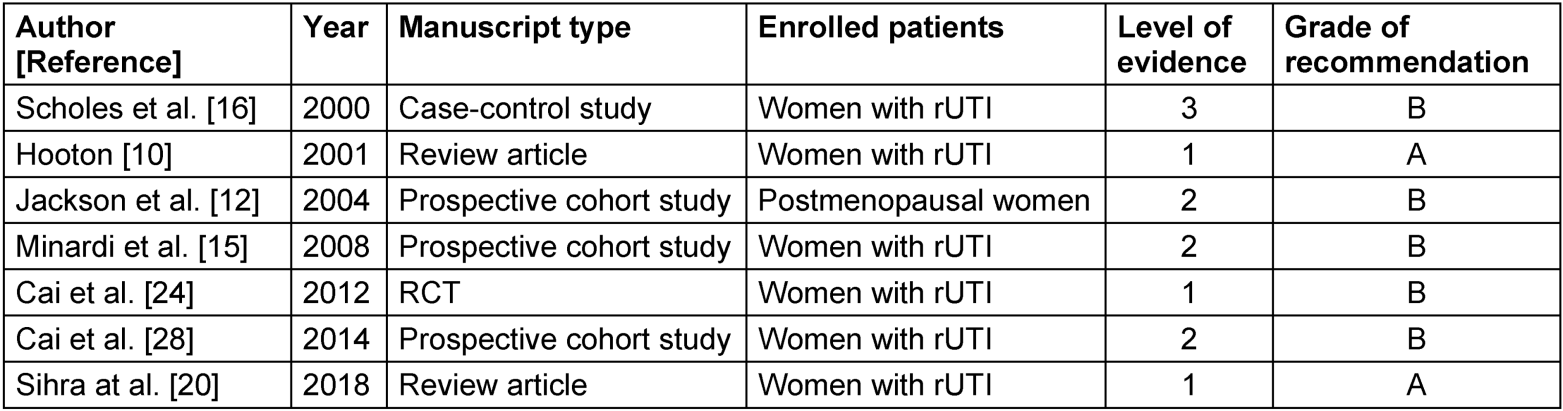
Studies evaluated after systematic review (1979–2019)
